# Establishment and validation of the cut-off values of estimated glomerular filtration rate and urinary albumin-to-creatinine ratio for diabetic kidney disease: A multi-center, prospective cohort study

**DOI:** 10.3389/fendo.2022.1064665

**Published:** 2022-12-12

**Authors:** Zhongai Gao, Yanjuan Zhu, Xiaoyue Sun, Hong Zhu, Wenhui Jiang, Mengdi Sun, Jingyu Wang, Le Liu, Hui Zheng, Yongzhang Qin, Shuang Zhang, Yanhui Yang, Jie Xu, Juhong Yang, Chunyan Shan, Baocheng Chang

**Affiliations:** ^1^ NHC Key Laboratory of Hormones and Development, Tianjin Key Laboratory of Metabolic Diseases, Chu Hsien-I Memorial Hospital & Tianjin Institute of Endocrinology, Tianjin Medical University, Tianjin, China; ^2^ Department of Epidemiology and Biostatistics, School of Public Health, Tianjin Medical University, Tianjin, China; ^3^ Department of Geriatrics, The Second Hospital of Tianjin Medical University, Tianjin, China; ^4^ Department of endocrinology, TEDA International Cardiovascular Disease Hospital, Tianjin, China

**Keywords:** diabetic kidney disease, estimated glomerular filtration rate, microalbuminuria, cardiovascular disease, diabetic metabolism

## Abstract

**Objective:**

We aimed to study the cut-off values of estimated glomerular filtration rate (eGFR) and the urinary albumin creatinine ratio (UACR) in the normal range for diabetic kidney disease (DKD).

**Methods:**

In this study, we conducted a retrospective, observational cohort study included 374 type 2 diabetic patients who had baseline eGFR ≥60 mL/min/1.73 m^2^ and UACR <30 mg/g with up to 6 years of follow-up. The results were further validated in a multi-center, prospective cohort study.

**Results:**

In the development cohort, baseline eGFR (AUC: 0.90, cut-off value: 84.8 mL/min/1.73 m^2^, sensitivity: 0.80, specificity: 0.85) or UACR (AUC: 0.74, cut-off value: 15.5mg/g, sensitivity: 0.69, specificity: 0.63) was the most effective single predictor for DKD. Moreover, compared with eGFR or UACR alone, the prediction model consisted of all of the independent risk factors did not improve the predictive performance (*P >*0.05). The discrimination of eGFR at the cut-off value of 84.80 mL/min/1.73 m2 or UACR at 15.5mg/g with the largest Youden’s index was further confirmed in the validation cohort. The decrease rate of eGFR was faster in patients with UACR ≥15.5mg/g (*P <*0.05). Furthermore, the decrease rate of eGFR or increase rate of UACR and the incidence and severity of cardiovascular disease (CVD) were higher in patients with eGFR ≤84.8 mL/min/1.73 m^2^ or UACR ≥15.5mg/g (*P <*0.05).

**Conclusions:**

In conclusion, eGFR ≤84.8mL/min/1.73 m^2^ or UACR ≥15.5mg/g in the normal range may be an effective cut-off value for DKD and may increase the incidence and severity for CVD in type 2 diabetic patients.

## 1 Introduction

Diabetic kidney disease (DKD) is a major microvascular complication of diabetes with high morbidity and mortality (1, 2), which are partly attributable to the lack of an early diagnosis of DKD. Therefore, the diagnosis of DKD has been the main focus of attention. Currently, an increasing number of studies have been conducted to identify novel biomarkers, such as protein, mRNA and microRNA, of early-stage renal injury (3, 4). However, owing to the low sensitivity and heterogeneous of these biomarkers, they are still far from clinical application. Thus, at present, eGFR and albuminuria are still used widely as golden diagnostic markers of DKD (5, 6). It was reported that low level of eGFR and high level of UACR in the normal range are risk factors for DKD. Whereas, the predictive power and the earlier cut-off value of the eGFR and UACR for DKD are largely unknown.

The decreased eGFR is defined as an indicator of DKD (3). The traditional view is that, after long-term exposure of microalbuminuria, which, is followed by a decline in GFR that ultimately leads to end-stage renal disease (7). However, in the U.K. Prospective Diabetes Study (UKPDS), of the patients who developed reduced eGFR, 61% did not have pre-existing albuminuria and 39% never developed albuminuria during the study (8). Thus, non-albuminuric renal impairment has become the prevailing DKD phenotype in patients with type 2 diabetes who have decreased eGFR (8–13). In The Kidney Disease: Improving Global Outcomes (KDIGO) guideline, an eGFR between 60 and 89 mL/min/1.73 m^2^ is considered to be a mild decrease; however, in the absence of evidence of kidney damage, neither of the GFR category G1 (≥90 mL/min/1.73 m^2^) nor and G2 (60–89 mL/min/1.73 m^2^) fulfil the criteria for chronic kidney disease (CKD) (14). Likewise, an eGFR <60 mL/min/1.73 m^2^ is the diagnostic criteria for DKD in the absence of evidence of kidney damage. However, in patients without evidence of kidney damage, the effect of eGFR in the normal range, especially in the range of 60-89 mL/min/1.73 m^2^ for predicting reduced eGFR is unclear.

Besides eGFR, microalbuminuria is also defined as an indicator of DKD. According to a prospective study with a follow-up period of 10 years by Mogensen in 1986, microalbuminuria (MAU) ≥30 mg/24 h is believed to be an early diagnostic marker indicates the optimal time for intervention (15, 16). However, even with positive intervention, approximately one third of patients with microalbuminuria will progress to macroalbuminuria, as reported from the Multifactorial Intervention for Patients with Type 2 Diabetes Study (17). This pattern is observed because the presence of microalbuminuria in patients with diabetes often implies that the kidneys have undergone different degrees of irreversible structural injuries. Furthermore, some studies have indicated that increased baseline UACR, even within the normal range, was a risk factor for DKD (18–21). According to research findings, participants with diabetes and a UACR of 10-30 mg/g had a 2.7-fold higher odds of developing albuminuria than those with UACR <5 mg/g (20). In addition, the KDOQI recommended a stratification of normal albuminuria to UACR <10 mg/g (optimal) and 10 ≤ UACR <30 mg/g (normal high limit) (14). But few studies have been conducted to determine the predictive power and the earlier cut-off value of UACR for predicting DKD.

Therefore, in this research, we aimed to study the predictive power and determine earlier cut-off values of eGFR and UACR in the normal range for predicting DKD, respectively, in patients with type 2 diabetes. In addition, in order to study the effect of the cut-off values we defined, we also studied the relationship between eGFR and UACR, and the relationship between the cut-off values and cardiovascular disease (CVD).

## 2 Methods

### 2.1 Study population

Two independent patient cohorts were used.

#### 2.1.1 Development cohort

**Figure 1:**
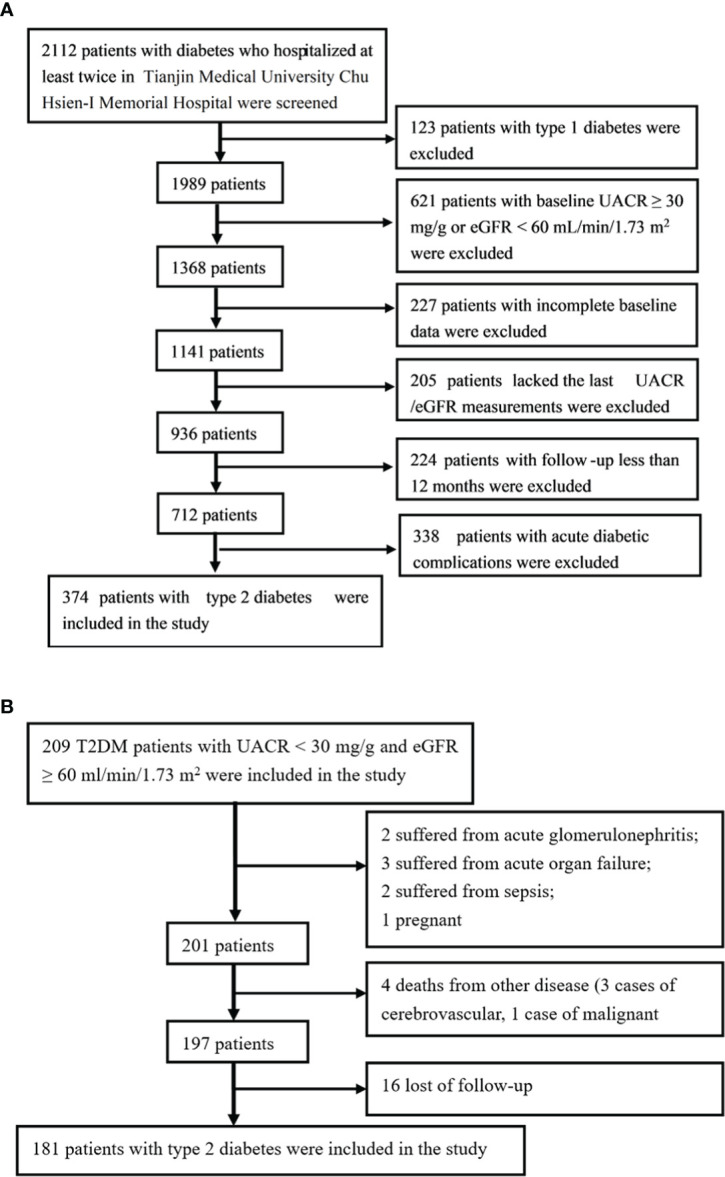
The flowcharts outlining selection of cohorts. **(A)** The flowchart outlining selection of the development cohort. **(B)** The flowchart outlining selection of the validation cohort.

This was a retrospective, observational cohort study. A total of 2112 diabetic patients who were hospitalised at least twice in Tianjin Medical University Chu Hsien-I Memorial Hospital (baseline period from July 2012 to August 2017) were screened for these inclusion criteria: eGFR >60 mL/min/1.73 m^2^ at baseline and normoalbuminuria (UACR <30 mg/g), age ≥18 years and follow-up duration >12 months. Because other kidney diseases may affect kidney function, induce albuminuria and increase the chance of hospitalisation, we excluded patients with a history of acute kidney injury, urinary calculi, chronic glomerulonephritis, IgA nephropathy, lupus nephritis, polycystic kidney disease, hypertensive nephropathy, gout-associated nephropathy, urinary tract infection, renal tubular injury, etc. After excluding 123 patients with type 1 diabetes, 621 patients with baseline eGFR <60 mL/min/1.73 m^2^ or UACR ≥30 mg/g, 227 patients with incomplete baseline data, 205 patients who lacked the last eGFR/AER/UACR measurements, 224 patients with a follow-up duration <12 months, and 338 patients with acute diabetes-related complications or serious infection, we included 374 patients with type 2 diabetes. A flowchart outlining the selection of study participants is shown in **Figure 1A**.

The outcomes were: (1) the occurrence of an eGFR decrease <60 mL/min/1.73 m^2^ for 3 months caused by diabetes, but without albuminuria at baseline and at the end of the follow-up; (2) the occurrence of albuminuria, including microalbuminuria and macroalbuminuria, defined as a UACR ≥30 mg/g for 3 months caused by diabetes.

#### 2.1.2 Validation cohort

A prospective cohort study was conducted in patients with type 2 diabetes to validate the cut-off value established in the development cohort. Patients enrolled in the study were randomly selected from three tertiary hospitals and prospectively followed up from July 2013 to July 2018. All of the patients had an eGFR ≥60 mL/min/1.73 m^2^ and UACR <30 mg/g at baseline.

The follow-up process for eGFR and UACR were as follows. Patients included in this study were required to undergo bi-annual re-examinations of their eGFR and UACR. Moreover, general indicators including body weight, blood pressure, HbA_1c_ and medications were recorded at each follow-up visit.

The exit criteria was specified as: (1) use of nephrotoxic drugs such as non-steroidal anti-inflammatory drugs during follow-up; (2) acute and chronic glomerulonephritis, urinary calculi, IgA nephropathy, lupus nephritis, polycystic kidney disease, hypertensive nephropathy, gout-associated nephropathy, urinary tract infection, renal tubular injury, sepsis, severe pneumonia, acute hepatitis, severe trauma, acute organ failure, tumour and any other severe diseases during follow-up; (3) pregnancy or childbirth during follow-up; and (4) death during follow-up. The flowchart is shown in **Figure 1B**.

The study endpoint was the detection of an eGFR <60 mL/min/1.73 m^2^ or UACR ≥30 mg/g during follow-up.

### 2.2 Data collection

Data on demographics and clinical measurements were collected from the medical records including age, sex, BMI, diabetes duration, blood pressure, and medication use. Direct ophthalmoscopy for diagnosing diabetic retinopathy (DR) was performed by an experienced ophthalmologist (no, 0; yes, 1). All blood samples were drawn from the patients after 12-hour overnight fasting. The routine investigations included serum total cholesterol (TC), triglyceride (TG), HDL, LDL, creatinine (SCr) and uric acid (SUA) that were measured using an AU5800 automatic biochemical analyser. HbA1c was measured using the HLC-723G8 HbA1c analyser. The Chronic Kidney Disease Epidemiology Collaboration (CKD-EPI) formula (22) was used to calculate the eGFR. The UACR was measured by immunoturbidimetry. The first-void midstream urine samples on two consecutive days were obtained to determine the level of UACR, and the mean value was included for analysis. All specimens were tested in the Department of Clinical Biochemical Laboratory at Tianjin Medical University Chu Hsien-I Memorial Hospital. CVD was diagnosed by coronary angiography defined as one or more diseased epicardial vessels with a diameter of more than 2 mm that had at least a 50% diameter stenosis.

### 2.3 Ethics statement

This study was approved by the Institutional Review Board of Tianjin Medical University Chu Hsien-I Memorial Hospital and Tianjin Institute of Endocrinology. Informed consent was obtained from all patients prior to study participation.

### 2.4 Statistical analyses

Quantitative data with normal and non-normal distribution are expressed as the mean ± SD and the median (first quartile, third quartile). Independent-sample *t*-test and nonparametric tests were used to analyse between-group differences for data with normal and non-normal distributions. All variables with a *P*-value <0.10 on univariate analysis were included in the Cox multivariate regression analysis to determine predictors and establish prediction models. The receiver operating characteristic (ROC) curve analysis (23) was used to evaluate the discrimination of the predictors and the prediction models. All sensitivity/specificity values were selected from the cut-off value with the largest Youden’s index. The calibration of the predictors was evaluated by the Hosmer–Lemeshow (H-L) test and *P >*0.05 indicates excellent goodness-of-fit. All of the above analyses were performed using SPSS software (SPSS, version 22, Chicago, USA). MedCalc Statistical Software (Medcalc, version 19, Ostend, Belgium) was used to compare the predictive power of the models. All statistical tests were two-tailed, and a *P-*value <0.05 was considered statistically significant.

## 3 Results

### 3.1 Assessment of the cut-off value of eGFR and UACR in predicting DKD in the development cohort

During an average 3-year follow-up duration, 8.5% (32/374) had reduced eGFR (<60 mL/min/1.73 m^2^), 19.8% (74/374) of the patients had albuminuria, and 1.6% (6/374) had both reduced eGFR and albuminuria.

#### 3.1.1 eGFR ≤84.8 mL/min/1.73 m^2^ was an earlier predictive cut-off value for DKD

During the average 3-year follow-up duration, 7% (26/368) of patients had reduced eGFR (eGFR <60 mL/min/1.73 m^2^) without albuminuria at baseline and at the end of the follow-up. No significant differences in the follow-up time, BMI, blood pressure, HbA_1c_, serum lipid, UACR and medications were observed between the patients with or without reduced eGFR. In patients with reduced eGFR, the age, sex, diabetes duration, SCr, SUA, baseline eGFR and percentage of DR were statistically different than those in patients without reduced eGFR (*P* < 0.05) (**Table 1**).

**Table 1:** Baseline characteristics of patients with T2DM according to the eGFR greater than or less than 60 ml/min/1.73 m^2^ in development cohort.

Variables	eGFR < 60 ml/min/1.73 m^2^		*P* value
	No	Yes
n	342	26	
Follow-up (months)	34.97 ± 14.81	31.82 ± 17.38	0.301
Age (years)	54.29 ± 10.35	60.50 ± 9.79	0.003
Female (%)	162 (47.4)	2 (7.7)	0.000
Diabetes duration (years)	6 (3, 12)	15 (5, 20)	0.002
BMI (kg/m^2^)	26.53 ± 3.53	27.22 ± 4.58	0.345
SBP (mmHg)	130 (120, 140)	135 (127, 146)	0.094
DBP (mmHg)	80 (70, 85)	80 (70, 90)	0.455
HbA1c (%)	8.50 (7.30, 9.90)	9.15 (6.80, 10.10)	0.489
TG (mmol/L)	1.63 (1.11, 2.54)	1.78 (1.19, 2.40)	0.535
HDL-C (mmol/L)	1.20 (1.10, 1.40)	1.30 (1.08, 1.50)	0.775
TC (mmol/L)	5.02 ± 1.12	5.16 ± 0.89	0.557
LDL-C (mmol/L)	3.12 ± 0.90	3.15 ± 0.74	0.888
SCr (umol/L)	60.47 ± 11.51	76.65 ± 8.28	0.000
SUA (umol/L)	301.47 ± 78.77	352.62 ± 69.22	0.002
Baseline eGFR (ml/min/1.73 m^2^)	101.18 ± 17.07	74.85 ± 11.63	0.000
Baseline UACR (mg/g)	14.58 (11.77, 18.25	14.38 (12.47, 19.35)	0.410
DR (n (%))	84 (24.6)	11 (42.3)	0.040
Oral diabetic medications (n (%))	338 (98.8)	26 (100)	0.538
Insulin (n (%))	197 (57.6)	11(42.3)	0.157
ACEI/ARB (n (%))	97 (28.4)	12 (46.2)	0.071
Statins (n (%))	88 (25.7)	6 (23.1)	0.802

Univariate Cox regression analysis showed that age, sex, diabetes duration, BMI, SBP, SUA, SCr, baseline eGFR and DR had *P*-values <0.1. As age, sex and SCr were involved in calculating eGFR, we only included the diabetes duration, BMI, SBP, SUA, baseline eGFR and DR to the multivariable Cox regression analysis, and the results indicated that the diabetes duration, SBP, baseline eGFR and DR were independent risk factors (**Table 2**).

**Table 2:** Univariate and multivariable Cox analysis for the risk factors of DKD.

	Univariate COX analysis				Multivariable Cox analysis			
	B	HR	95% CI	*P* value	B	HR	95% CI	*P* value
Baseline eGFR (ml/min/1.73 m^2^)	-0.100	0.905	0.876-0.934	0.000	-0.118	0.888	0.855-0.922	0.000
Diabetes duration (years)	0.107	1.113	1.055-1.173	0.000	0.063	1.065	1.006-1.128	0.030
SBP (mmHg)	0.021	1.021	0.999-1.045	0.066	0.035	1.035	1.008-1.063	0.011
DR	1.350	3.857	1.669-8.914	0.002	1.211	3.355	1.359-8.285	0.009
Age (years)	0.062	1.064	1.022-1.108	0.003					
Gender	-2.173	0.114	0.030-0.438	0.002					
BMI (kg/m^2^)	0.098	1.103	1.005-1.210	0.040					
DBP (mmHg)	0.028	1.028	0.990-1.067	0.153					
HbA1c (%)	0.127	1.135	0.925-1.392	0.224					
TG (mmol/L)	-0.068	0.934	0.718-1.215	0.613					
HDL-C (mmol/L)	-0.803	0.448	0.115-1.751	0.248					
TC (mmol/L)	-0.001	0.999	0.690-1.446	0.996					
LDL-C (mmol/L)	-0.018	0.982	0.620-1.556	0.939					
SUA (umol/L)	0.008	1.008	1.004-1.013	0.001					
SCr (umol/L)	0.109	1.115	1.076-1.156	0.000					
Baseline UACR (mg/g)	0.038	1.039	0.971-1.110	0.268					

**Figure 2:**
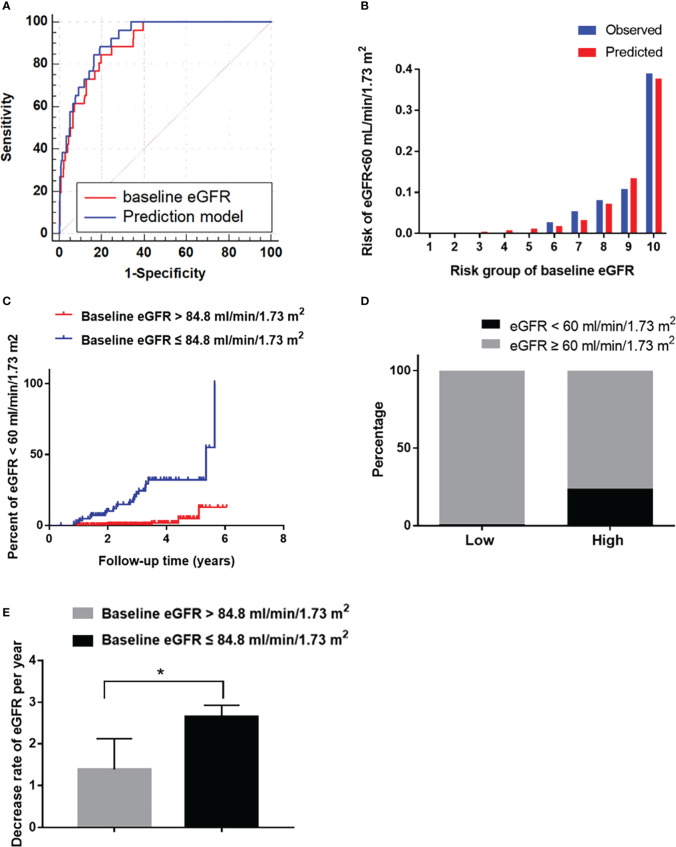
The performance of the baseline eGFR in predicting reduced eGFR in the development cohort. **(A)** Receiver operating characteristic curve for the baseline eGFR and the prediction models. The area under the curve (AUC) and its 95% CI were 0.90 (0.85–0.96) for the baseline eGFR and 0.91 (0.87-0.96) for the prediction model; The discriminative power of baseline eGFR vs prediction model (*P*=0.381). **(B)** Hosmer-Lemeshow (H-L) test for the calibration of the baseline eGFR (*P*=0.87). **(C)** Kaplan-Meier curve of reduced eGFR end point for the low-risk (eGFR >84.8 mL/min/1.73 m^2^) and high-risk (60 ≤ eGFR ≤ 84.8 mL/min/1.73 m^2^) groups stratified according to the cut-off value. The HR (95% CI) was 17.47 (6.00-50.81) (*P*<0.001) in the high-risk group. **(D)** Prevalence of reduced eGFR in the low-risk (eGFR >84.8 mL/min/1.73 m^2^) and high-risk (60 ≤ eGFR ≤ 84.8 mL/min/1.73 m^2^) groups. **(E)**: The decrease rate of eGFR per year in the low-risk (eGFR >84.8 mL/min/1.73 m^2^) and high-risk (60 ≤ eGFR ≤ 84.8 mL/min/1.73 m2) groups. *P < 0.05.

The predictive power of the risk factors and the prediction model included all of the risk factors were evaluated. The AUC of diabetes duration, SBP and DR in predicting a reduced eGFR was 0.68 (*P*=0.002), 0.59 (*P*=0.11) and 0.59 (*P*=0.12), respectively (**Table 3**). The baseline eGFR was the most effective independent predictor for reduced eGFR, with great predictive power (AUC: 0.90, cut-off value: 84.80, sensitivity: 0.80, specificity: 0.85) and goodness-of-fit (*P*=0.98, **Figure 2B**). Furthermore, compared with baseline eGFR alone, the prediction model established with all of the independent risk factors including diabetes duration, SBP and DR and eGFR did not improve the predictive performance (AUC 0.91, *P*=0.381) (**Figure 2A**).

**Table 3:** The discriminative power of the predictors and the prediction model in prediting DKD.

Cut-off value of eGFR (mL/min/1.73 m^2^)	Sensitivity	Specificity	Youden Index
95.0	0.90	0.65	0.55
90.0	0.85	0.74	0.59
**84.8**	**0.80**	**0.85**	**0.65 (the largest)**
80.0	0.65	0.88	0.53
75.0	0.55	0.93	0.48

Furthermore, in order to estimate the optimal cut-off value, we assessed the sensitivity, specificity and Youden Index of eGFR at several cut-off values. In the results, 84.8 was selected as the optimal cut-off value based on the maximum value of the Youden Index, and the sensitivity and specificity were 0.80 and 0.85, respectively, at 84.8 mL/min/1.73 m^2^ of eGFR. Besides, the sensitivity, specificity and Youden Index at different cut-off value are shown in **Table 4**.

**Table 4:** Sensitivity, specificity and youden index of the eGFR at different cut-off values.

	AUC	Cut-off value	Sensitivity	Specifity	P value	95% CI
Diabetes duration (years)	0.68	/	/	/	.002	0.56-0.81
SBP (mmHg)	0.59	/	/	/	0.11	0.49-0.70
DR	0.59	/	/	/	0.12	0.47-0.71
Baseline eGFR (ml/min/1.73 m^2^)	0.90	84.80	0.80	0.85	0.000	0.85-0.96
Prediction model*	0.91	/	/	/	0.000	0.86-0.96

*The prediction model for reduced eGFR consists of all of the independent risk factors including diabetes duration, SBP, DR, baseline eGFR. /, null term.

Then it was divided into low-risk (eGFR >84.8 mL/min/1.73 m^2^) and high-risk (60 ≤ eGFR ≤ 84.8 mL/min/1.73 m^2^) groups. The HR (95% CI) were 17.47 (6.00–50.81) in the high-risk group (*P*<0.001, **Figure 2C**). The incidence of reduced eGFR in the low-risk and high-risk groups were 1% (4/276) and 24% (22/92), respectively (**Figure 2D**). The decrease rate of eGFR in the high-risk group was significantly higher than in low-risk group (*P*<0.05, **Figure 2E**).

#### 3.1.2 UACR ≥15.5 mg/g was an earlier predictive cut-off value for DKD

During an average 3-year follow-up duration, 19.8% (74/374) of patients had albuminuria. Among them, 18.2% (68/374) had microalbuminuria and 1.6% (6/374) had macroalbuminuria. No significant differences in the follow-up time, age, sex, diabetes duration, BMI, blood pressure, HbA1c, serum lipids, SCr, SUA, eGFR, and medication use were observed between patients with or without albuminuria. In patients with albuminuria, the baseline UACR (median: 19.52, IQR: 14.53–28.70) was significantly higher than that in patients without albuminuria (median: 14.00; IQR: 11.50–17.50). The percentage of DR patients with albuminuria (43.2%) was significantly higher than that of patients without albuminuria (21.0%) (**Table 5**).

**Table 5:** Baseline characteristics of patients with T2DM according to the presence or absence of albuminuria in development cohort.

Variables	Total	Any albuminuria		*P* value
		No	Yes
n	374	300	74	
Follow-up (months)	35.22 ± 15.13	34.35 ± 14.81	36.42 ± 15.74	0.29
Age (years)	54.72 ± 10.42	54.66 ± 10.35	54.96 ± 10.77	0.83
Female (%)	170 (45.5)	135 (45)	29 (39)	0.58
Diabetes duration (years)	7 (3, 12)	7 (3, 13)	8 (3, 10)	0.78
BMI (kg/m^2^)	26.58 ± 3.61	26.66 ± 3.63	26.26 ± 3.56	0.39
SBP (mmHg)	130 (120, 140)	130 (120, 140)	130 (120, 140)	0.32
DBP (mmHg)	80 (70, 85)	80 (70, 85)	80 (70, 90)	0.84
HbA1c (%)	8.60 (7.30, 9.93)	8.50 (7.30, 9.80)	8.90 (7.35, 10.50)	0.131
TG (mmol/L)	1.63 (1.12, 2.53)	1.63 (1.10, 2.52)	1.66 (1.17, 2.77)	0.454
HDL-C (mmol/L)	1.20 (1.10, 1.40)	1.20 (1.10, 1.40)	1.20 (1.08, 1.40)	0.195
TC (mmol/L)	5.03 ± 1.10	5.01 ± 1.05	5.11 ± 1.34	0.548
LDL-C (mmol/L)	3.13 ± 0.89	3.09 ± 0.84	3.27 ± 1.05	0.111
SCr (umol/L)	61.59 ± 12.03	61.60 ± 12.13	61.56 ± 11.73	0.979
SUA (umol/L)	304.96 ± 79.13	301.51 ± 80.23	318.56 ± 73.61	0.098
Baseline eGFR (ml/min/1.73 m^2^)	99.35 ± 18.03	99.48 ± 18.24	98.85± 17.28	0.789
Baseline UACR (mg/g)	14.56 (11.86, 18.49)	14.00 (11.50, 17.50)	19.52 (14.53, 28.70)	< 0.001
DR (n (%))	95 (25.8)	63 (21.0)	32 (43.2)	< 0.001
Oral diabetic medications (n (%))	364 (98.9)	297 (99.0)	72 (97.3)	0.25
Insulin (n (%))	208 (56.5)	163 (54.3)	45 (60.8)	0.32
ACEI/ARB (n (%))	109 (29.6)	89 (29.7)	20 (27.0)	0.65
Statins (n (%))	94 (25.5)	73 (24.3)	21 (28.4)	0.47

We further verified the risk factors for albuminuria. Univariate analysis showed that the SBP, DBP, HbA1c, HDL-C, SUA, and baseline UACR had P-values <0.1. In multivariable Cox regression analysis, we determined that the HbA1c, HDL-C, baseline UACR, and DR were independent risk factors (**Table 6**).

**Table 6:** Univariate and multivariable Cox analysis for the risk factors of DKD.

	Univariate COX analysis				Multivariable Cox analysis			
	B	HR	95% CI	*P* value	B	HR	95% CI	*P* value
Baseline UACR (mg/g)	0.118	1.125	1.087-1.165	0.000	0.109	1.116	1.077-1.156	0.000
DR	1.635	5.128	3.064-8.584	0.000	1.302	3.677	2.167-6.238	0.000
HbA1c (%)	0.195	1.215	1.078-1.369	0.001	0.175	1.192	1.040-1.366	0.012
HDL-C (mmol/L)	-1.399	0.247	0.103-0.593	0.002	-1.109	0.330	0.149-0.731	0.006
Age (years)	0.007	1.007	0.985-1.030	0.517				
Gender	-0.246	0.782	0.489-1.249	0.304				
Diabetes duration (years)	0.007	1.007	0.970-1.046	0.705				
TG (mmol/L)	0.065	1.067	0.984-1.158	0.115				
BMI (kg/m^2^)	0.049	1.050	0.989-1.116	0.112				
SBP (mmHg)	0.015	1.015	1.001-1.029	0.038				
DBP (mmHg)	0.021	1.021	0.998-1.045	0.075				
TC (mmol/L)	-0.071	0.932	0.745-1.166	0.536				
LDL-C (mmol/L)	0.143	1.154	0.888-1.500	0.284				
SUA (umol/L)	0.003	1.003	1.000-1.005	0.083				
SCr (umol/L)	-0.005	0.995	0.976-1.014	0.601				
Baseline eGFR (ml/min/1.73 m^2^)	-0.001	0.999	0.986-1.012	0.889				

**Figure 3:**
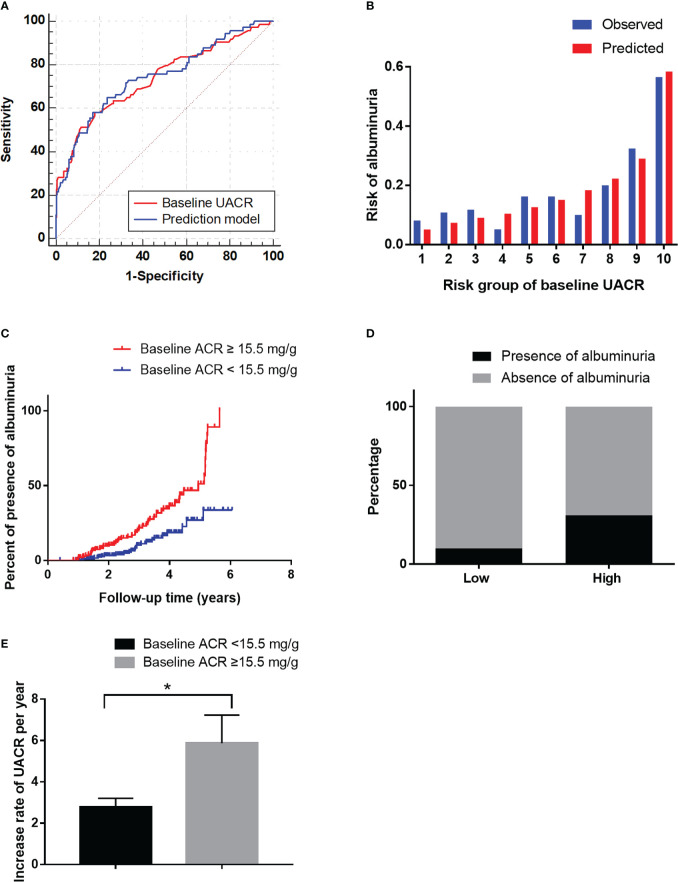
The performance of the baseline UACR in predicting albuminuria in the development cohort. **(A)**: Receiver operating characteristic curve for the baseline UACR and the prediction models. The area under the curve (AUC) and its 95% CI were 0.74 (0.67–0.81) for the baseline UACR and 0.74 (0.68-0.81) for the prediction model. The discriminative power of baseline UACR vs the prediction model (P=0.465). **(B)**: Hosmer-Lemeshow (H-L) test for the calibration of the baseline UACR (P=0.22). **(C)**: Kaplan-Meier curve of albuminuria end point for the low-risk (UACR < 15.5 mg/g) and high-risk groups (30 > UACR ≥ 15.5 mg/g) according to the cut-off value. The HR (95% CI) was 1.66 (1.31-2.10) (P < 0.001) in the high-risk group. **(D)**: Prevalence of albuminuria in the low-risk (UACR <15.5 mg/g) and high-risk (30 > UACR ≥ 15.5 mg/g) groups. **(E)**: The increase rate of UACR per year in the low-risk (UACR < 15.5 mg/g) and high-risk groups (30 > UACR ≥ 15.5 mg/g) according to the cut-off value. *P < 0.05.

Furthermore, we evaluated the predictive power of the risk factors and the prediction model included all of the independent risk factors. The AUC of HbA1c, HDL-C and DR were 0.56 (P=0.131), 0.45 (P=0.199) and 0.61 (P=0.003) for predicting albuminuria (**Table 7**). Baseline UACR was the most effective single predictor for predicting albuminuria (AUC: 0.74, cut-off value: 15.5, sensitivity: 0.69, specificity: 0.63), and it had a goodness-of-fit in predicting albuminuria (P=0.22, **Figure 3B**). Moreover, compared with UACR alone, the prediction model consisted of all of the independent risk factors including HbA1c, HDL-C, DR, baseline UACR did not improve the predictive performance (AUC 0.74, P=0.465, **Figure 3A**).

**Table 7:** The discriminative power of the predictors and the prediction model in predicting DKD.

	AUC	Cut-off value	Sensitivity	Specificity	*P* value	95% CI
HbA1c (%)	0.56	/	/	/	0.131	0.48-0.63
HDL-C (mmol/L)	0.45	/	/	/	0.199	0.38-0.53
DR	0.61	/	/	/	0.003	0.54-0.69
Baseline UACR (mg/g)	0.74	15.5	0.69	0.63	0.000	0.67-0.81
Prediction model*	0.74	/	/	/	0.000	0.68-0.81

*The prediction model for albuminuria consists of all of the independent risk factors including HbA1c, HDL-C, DR and baseline UACR. /, null term.

Furthermore, in order to estimate the optimal cut-off value, we assessed the sensitivity, specificity and Youden Index of UACR at several cut-off values. In the results, 15.5 was selected as the optimal cut-off value based on the maximum value of the Youden Index, and the sensitivity and specificity were 0.69 and 0.63, respectively. Besides, the sensitivity, specificity and Youden Index at different cut-off values are shown in **Table 8**.

**Table 8:** Sensitivity, specificity and youden index of the UACR at different cut-off values.

Cut-off value ofUACR (mg/g)	Sensitivity	Specificity	Youden Index
5.0	1	0.02	0.02
10.0	0.93	0.16	0.09
**15.5**	**0.69**	**0.63**	**0.32(the largest)**
20.0	0.31	0.89	0.20
25.0	0.28	0.96	0.24

Then it was divided into low-risk (UACR <15.5 mg/g) and high-risk (15.5 < UACR ≤ 30 mg/g) groups based on the cut-off value. The HR (95% CI) were 1.66 (1.31–2.10) (P<0.001) in the high-risk group (**Figure 3C**). The incidence of albuminuria in the low-risk and high-risk groups were 10%(22/211) and 32% (52/163), respectively (**Figure 3D**). The increase rate of UACR in the high-risk group was significantly higher than in low-risk group (P<0.05, **Figure 3E**).

### 3.2 Validation of the cut-off value of eGFR ≤ 84.8 mL/min/1.73 m^2^ and UACR ≥15.5 mg/g in predicting DKD

Of the 209 participants in this study, 181 patients were followed up successfully for 36 (24–72) months; 24 patients (8.52%) exited the follow-up, including 4 deaths from other diseases (3 cases of cerebrovascular diseases, 1 case of malignant tumour), 1 pregnancy, 2 cases of acute glomerulonephritis, 2 cases of sepsis, 3 cases of acute organ failure, and 16 loss to follow-up. A flowchart outlining the patient disposition in the study is shown in **Figure 1B**.

During an average follow-up duration of 3 years, 17.1% (31/181) of patients had albuminuria, 13.8% (25/181) had reduced eGFR, and 2.2% (4/181) had both reduced eGFR and albuminuria.

#### 3.2.1 Validation of the cut-off value of eGFR ≤ 84.8 mL/min/1.73 m^2^ in predicting DKD and the relationship between eGFR and cardiovascular disease

After an average follow-up of 3 years, 11.9% (21/177) of patients had reduced eGFR (eGFR <60 mL/min/1.73 m^2^) without albuminuria at baseline and at the end of the follow-up.

**Figure 4:**
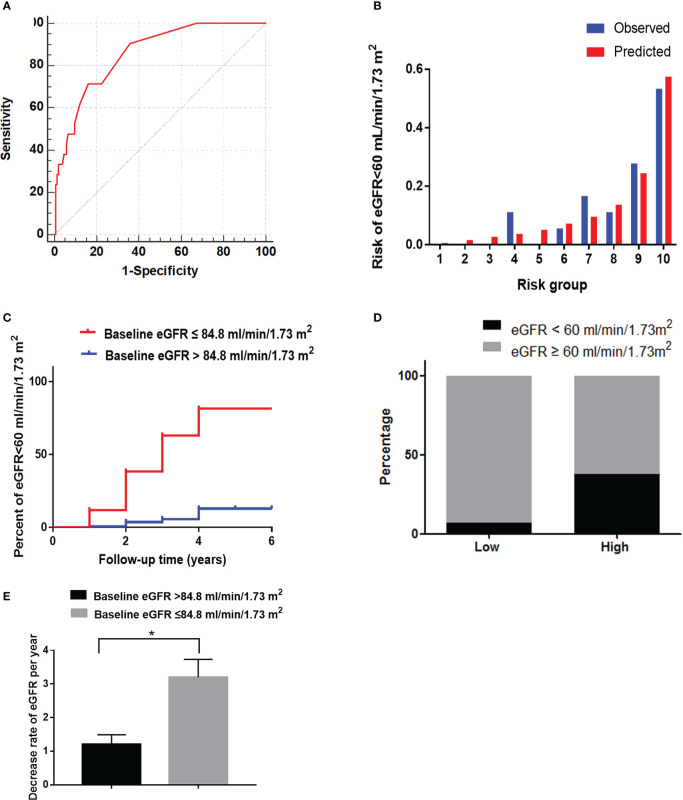
The performance of the baseline eGFR in predicting reduced eGFR in the validation cohort. **(A)** Receiver operating characteristic curve of baseline eGFR in predicting reduced eGFR. The area under the curve (AUC) and its 95%CI were 0.85(0.76-0.93). The sensitivity and specificity were 0.85 and 0.72, respectively, at 84.8 mL/min/1.73 m^2^ of eGFR. **(B)** Hosmer-Lemeshow (H-L) test for the calibration of the baseline eGFR(*P*=0.17). **(C)** Kaplan-Meier curve of reduced eGFR end point for the low-risk (eGFR >84.8 mL/min/1.73 m^2^) and high-risk (60 ≤ eGFR ≤ 84.8 mL/min/1.73 m^2^) groups. The HR (95% CI) was 15.84 (4.04-24.00) (*P* < 0.001) in the high-risk group. **(D)** Prevalence of reduced eGFR in the two risk groups. **(E)** The decrease rate of eGFR per year in the low-risk (eGFR > 84.8 mL/min/1.73 m^2^) and high-risk (60 ≤ eGFR ≤ 84.8 mL/min/1.73 m2) groups. *P < 0.05.

The baseline characteristics are listed in **Supplementary Table 2**. The AUC of the baseline eGFR was 0.85 (0.76-0.93) in the validation cohort according to the predictive probability of the development cohort. With a baseline eGFR of 84.8 mL/min/1.73 m^2^, the sensitivity and specificity were 0.85 and 0.72, respectively (**Figure 4A**). The predictor of baseline eGFR had a goodness-of-fit (*P*=0.17, **Figure 4B**). Moreover, the HR (95% CI) was 15.84 (4.04–24.00) (*P*<0.001) in the high-risk group (**Figure 4C**). The incidence of reduced eGFR in the low-risk and high-risk groups were 7% (11/159) and 39% (7/18), respectively (**Figure 4D**). The increase rate of UACR in the high-risk group was significantly higher than in low-risk group (*P*<0.05, **Figure 4E**).

**Figure 5:**
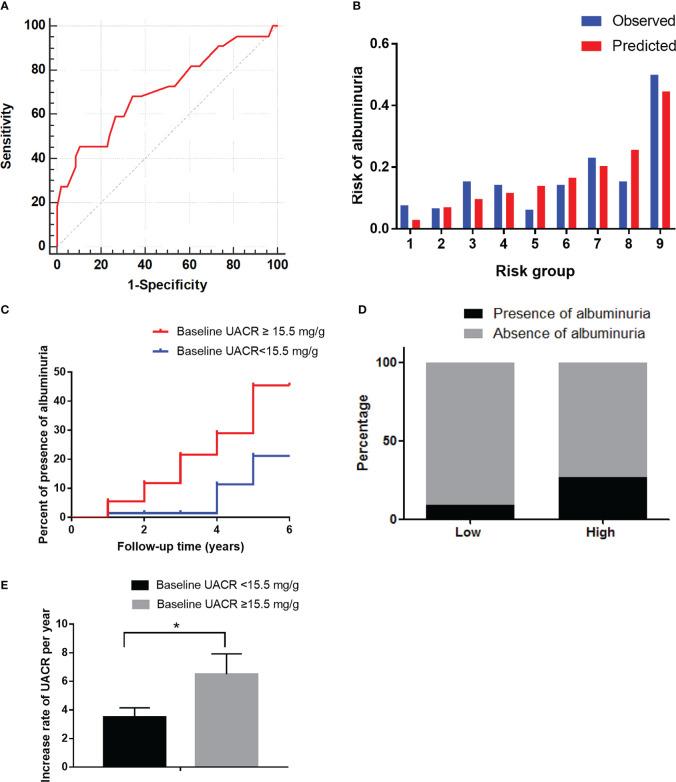
The performance of the baseline UACR in predicting albuminuria and baseline eGFR in predicting reduced eGFR in the validation cohort. **(A)**: Receiver operating characteristic curve of baseline UACR in predicting albuminuria. The area under the curve (AUC) and its 95% CI were 0.71(0.58-0.84). The sensitivity and specificity were 0.68 and 0.66, respectively, at 15.5mg/g of UACR. **(B)**: Hosmer-Lemeshow (H-L) test for the calibration of the baseline UACR (P=0.95). **(C)**: Kaplan-Meier curve of albuminuria end point for the low-risk (UACR < 15.5 mg/g) and high-risk (30 > UACR ≥ 15.5 mg/g) groups. The HR (95% CI) was 2.78 (1.31-9.28) (P = 0.035) in the high-risk group. **(D)**: Prevalence of albuminuria in the two risk groups. **(E)**: The increase rate of UACR per year in the low-risk (UACR < 15.5 mg/g) and high-risk groups (30 > UACR ≥ 15.5 mg/g) according to the cut-off value. *P < 0.05.

In the validation cohort, the incidence of CVD in the high-risk group of eGFR (17.4%) was significantly higher than in the low-risk group (7.4%) (**Figure 5A**, P<0.05). The lesion vessel number was significantly higher in the high-risk group of the eGFR than in the low-risk group (**Figures 5C, D**, P<0.05).

#### 3.2.2 Validation of the cut-off value of UACR ≥15.5 mg/g in predicting DKD and the relationship between UACR and cardiovascular disease

After an average 3-year follow-up duration, 17.1% of patients had albuminuria. Among them, 15.5% (28/181) had microalbuminuria and 1.7% (3/181) had macroalbuminuria.

**Figure 6:**
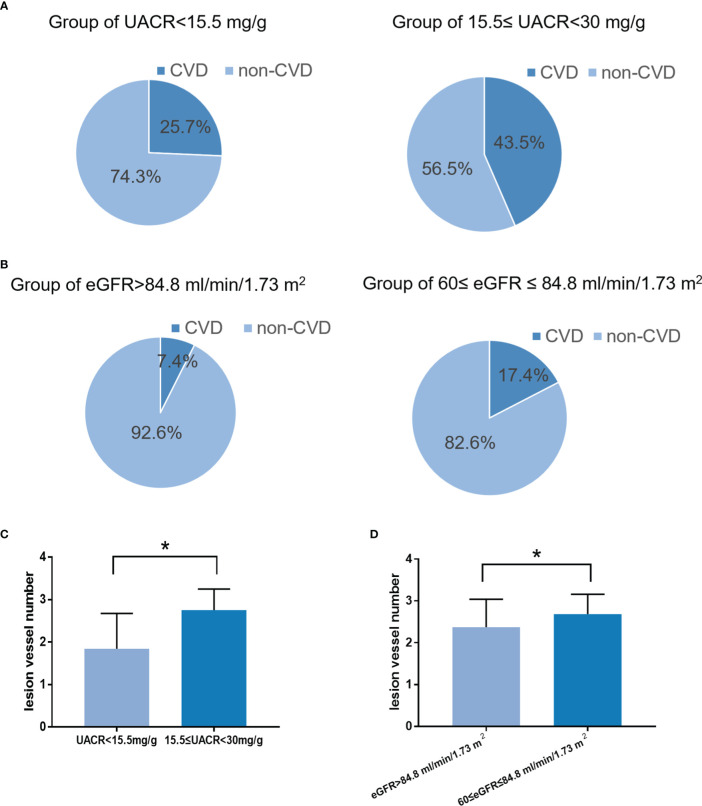
The incidence and severity of CVD in the low-risk and high-risk groups. **(A)**: The incidence and severity of CVD in the low-risk and high-risk groups according to the cut-off value of the UACR (*P < *0.05). **(B)**: The incidence and severity of CVD in the low-risk and high-risk groups according to the cut-off value of the eGFR (*P < *0.05). **(C)**: The severity of CVD in the low-risk and high-risk groups according to the cut-off value of the UACR (*P < *0.05). **(D)**: The severity of CVD in the low-risk and high-risk groups according to the cut-off value of the eGFR (*P < *0.05). *P < 0.05.

The baseline characteristics are shown in **Supplementary Table 1**. The AUC of the baseline UACR in the validation cohort was 0.71 (0.58–0.84) according to the predictive probability of the development cohort. At a UACR of 15.5 mg/g, the sensitivity and specificity were 0.68 and 0.66, respectively (**Figure 6A**). The predictor of baseline UACR had a goodness-of-fit (P=0.95, **Figure 6B**). Furthermore, the HR (95% CI) was 2.78 (1.31–9.28) (P=0.035) in the high-risk group (**Figure 6C**). The incidence of albuminuria in the low-risk and high-risk groups were 10% (10/103) and 27% (21/78), respectively (**Figure 6D**). The increase rate of UACR in the high-risk group was significantly higher than in low-risk group (P<0.05, **Figure 56**).

In the validation cohort, the incidence of CVD in the high-risk group of UACR (43.5%) was significantly higher than in the low-risk group (25.7%) (**Figure 5B**, P<0.05). The lesion vessel number was significantly higher in the high-risk group of the UACR than in the low-risk group (**Figure 5C, D**, P<0.05).

### 3.3 The relationship between eGFR and UACR

In the validation cohort, we further investigated the relationship between eGFR and UACR at the cut-off value metioned above. In the subgroup of eGFR ≤ 84.80 ml/min/1.73 m^2^, the endpoint eGFR was lower and the decline rate of eGFR was faster in the group of baseline UACR ≥ 15.5 mg/g than in the group of baseline UACR < 15.5 mg/g. In the subgroup of eGFR > 84.80 ml/min/1.73 m^2^, the eGFR and the decline rate of eGFR was not statistically different between the two groups (**Table 9**). Besides, the endpoint UACR had no significant difference in patients with eGFR lower or higher than 84.80 ml/min/1.73 m^2^ (**Supplementary Table 3**).

**Table 9:** The subgroup analysis of eGFR and eGFR decline rate in the normal and high-normal group of UACR according to the cut-off value of 15.5mg/g.

	Baseline eGFR > 84.80 mL/min/1.73 m^2^			Baseline eGFR ≤ 84.80 mL/min/1.73 m^2^		
Indicators	Baseline ACR <15.5	Baseline ACR ≥ 15.5	*P*	Baseline ACR <15.5	Baseline ACR ≥ 15.5	*P*
Baseline eGFR	103.65 ± 16.93	99.80 ± 15.87	0.11	76.36 ± 7.65	75.38 ± 7.37	0.63
Endpoint eGFR	103.08 ± 16.88	99.91 ± 15.90	0.17	77.94 ± 17.10	69.45 ± 15.06	0.04
eGFR decline rate (per month)	0.13 (0.01, 0.33)	0.16 (0.02, 0.44)	0.15	-0.11 (-0.41, 0.13)	0.12 (-0.05, 0.41)	0.02

## 4 Discussion

In this study, we identified that the eGFR and UACR in the normal range had the most predictive power for predicting DKD. The prediction models did not improve significantly by addition of further variables. Furthermore, we defined that eGFR at 84.8 mL/min/1.73 m^2^ or UACR at 15.5 mg/g with the largest Youden’s index were an earlier cut-off value for DKD. Patients with eGFR at 60–84.8 mL/min/1.73 m^2^ or UACR at 15.5-30 mg/g were more likely to develop DKD than patients with eGFR >84.8 mL/min/1.73 m^2^ or UACR <15.5 mg/g. Patients who had eGFR ≤84.80 ml/min/1.73 m^2^ or UACR ≥15.5 mg/g may had higher incidence and severity for CVD.

It was reported that eGFR and albuminuria were the most important factors to predict onset and progression of early CKD in individuals with type 2 diabetes, and inclusion of demographic, clinical, and other laboratory predictors barely improved predictive performance (24). Consistent with this study, the results in our study indicated that baseline eGFR was effective enough to predict DKD, and UACR was effective enough to predict DKD. In addition, adding other risk factors into the predictive model will not substantially improve risk prediction. This by no means negates the importance of the other risk factors such as hyperglycemia (25–27) and hyperlipidemia (28) as modifiable factors in the progression of DKD, because ability to predict the progression of the disease should not be equated with causality. On the contrary, it provides the simplest and most effective way to predict DKD.

The eGFR is the most important indicator that reflects renal function. Once the process of renal function decline begins, it will irreversibly progress to end-stage renal disease. Recent studies have shown that the decreased eGFR within the normal range may be a risk factor for DKD (29, 30). However, as the single predictor, the predictive performance of baseline eGFR was unclear. In this study, all of the patients had normal albuminuria at baseline, and in order to strictly exclude the patients with pre-existing kidney damage, we excluded patients with UACR ≥30 mg/g at the end of the follow-up. Our results demonstrated that the baseline eGFR as a single predictor is effective enough to identify patients at high risk for DKD with an AUC of 0.91. Furthermore, we determined the optimal cut-off value of eGFR at 84.8 mL/min/1.73 m^2^. In the validation cohort, at the eGFR of 84.8 mL/min/1.73 m^2^, the sensitivity and specificity were 85% and 72%, respectively. This added to the evidence base that the eGFR cut-off value of 84.8 mL/min/1.73 m^2^ was effective enough to distinguish high-risk from low-risk patients. Compared with participants in the low-risk group, those in the high-risk group had a 15.84-fold increase in the odds of DKD, and the decrease rate of eGFR was higher in patients with eGFR ≤84.80 ml/min/1.73 m^2^ than in patients with eGFR >84.80 ml/min/1.73 m2. Considering that eGFR is strongly influenced by age, we divided our patients into four groups according to quartiles: >61, 55-61, 48-55, or <48. We found that in each group, the cut-off of 84.8 had very consistent sensitivity and specificity. In addition, we included age in the multivariate COX regression analysis, and found that the inclusion of age either in the prediction model that included all risk factors with statistical differences or in the models that only included age and eGFR, could not improve the predictive value of the model. All these results support that the cut-off value is reliable across age. We also included gender into multivariable Cox analysis, but no statistical difference was found. Therefore, we believe that the cut-off value of eGFR at 84.8 mL/min/1.73 m^2^ can effectively predict the development of DKD. At the same time, we calculated the difference in eGFR between the groups at baseline and at the end of follow-up, and the results showed that patients with eGFR ≤84.8 had a greater eGFR decline than those with eGFR >84.8 even after the correction of follow-up time. These results suggest that patients with eGFR ≤ 84.8 have a faster decline of renal function. Thus we conclude that in the absence of albuminuria and other evidence of kidney damage, the progressive decline in renal function may already exist in patients with an eGFR of 60–84.8 mL/min/1.73 m^2^.

It is well known that hyperfiltration is one major feature of early DKD. Besides, in a follow-up study of patients with diabetic nephropathy, researchers at the Joslin Diabetes Center found that there was a progressive decline in eGFR before reaching the stage of less than 60 (31). Therefore, both hyperfiltration and a mild decrease in the normal range of eGFR may predict DKD. They may exist in different periods before the onset of DKD. In a meta-analysis including 23 studies, it was found that patients with hyperfiltration may need up to 10 years to develop DKD (32); However, in our study, the average time for patients with eGFR ≤84.80 ml/min/1.73 m^2^ to develop DKD is about 3 years. Therefore, eGFR ≤84.80 ml/min/1.73 m^2^ may be a more recent predictor of DKD compared with hyperfiltration. In addition, it is currently believed that there is a considerable proportion of non-albuminuria DKD in patients with DKD. These patients do not necessarily experience the classic Mogensen staging: hyperfiltration phase, intermittent microalbuminuria, persistent microalbuminuria phase, macroalbuminuria phase and failure of kidney function, and they may develop directly into the decline of renal function. Therefore, our study have suggested that eGFR ≤84.80 ml/min/1.73 m^2^ may provide a new clue for those who are at increased risk of developing non-albuminuria DKD.

UACR is a widely used indicator for diagnosing early DKD (33, 34). Previous studies have demonstrated that an increased baseline UACR, even within the normal range, was a risk factor for developing albuminuria (18–21). Participants with diabetes and a UACR of 10-30 mg/g had a 2.7-fold higher odds of developing albuminuria than those with UACR <5 mg/g (20). In addition, the KDOQI recommended a stratification of normal albuminuria to UACR <10 mg/g (optimal) and 10 ≤ UACR <30 mg/g (normal high limit) (14). Thus higher UACR in the normal range is indeed a risk factor for DKD. In this study, we assessed the predictive power of the baseline UACR and found that it was the most effective single predictor for predicting DKD with an AUC of 0.74. Furthermore, we determined that the optimal cut-off value of UACR is 15.5 mg/g. In the validation cohort, at an UACR cut-off value of 15.5 mg/g, the sensitivity and specificity were 0.68 and 0.66, respectively. In addition, compared with participants in the low-risk group, those in the high-risk groups had 2.78 fold increases in the odds of DKD, and the increase rate of UACR was higher in patients with UACR ≥15.5 mg/g than in patients with UACR <15.5 mg/g. Therefore, UACR at 15.5 mg/g may be a simple and effective cut-off value to distinguish high-risk patients from other populations. The increase of UACR in the normal range may reflect earlier abnormalities of glomerular haemodynamics and permselectivity (19) or the reabsorption dysfunction of the renal tubules (35). On the other hand, albuminuria may not simply be an indicator of damage in the glomerular filtration barrier, or as a predictor of DKD progression, because albuminuria in itself can be toxic to the kidney and affect pathological processes (36), such as causing tubulointerstitial inflammation, oxidative stress, fibrosis and tubular cell injury and death by activating a series of signalling pathways in proximal tubular cells (37–39). Therefore, the early prevention and treatment of UACR may prevent associated tubular injury and delay the progression of albuminuria.

It has been reported that elevated ACR, even within the normal range, is associated with a faster decline in eGFR in diabetic patients (40). Consistent with the previously reported literature, in this study, it was found that in the subgroup of the baseline eGFR lower than 84.80 ml/min/1.73 m^2^, the level of eGFR was lower and the decline rate of eGFR was faster in the patients with UACR ≥15.5 mg/g than in patients with UACR <15.5 mg/g. Thus we concluded that on the basis of eGFR at lower level in the normal range, UACR ≥ 15.5 mg/g may effectively indicate faster decline in eGFR.

The eGFR and UACR are independent predictors of cardiovascular events in type 2 diabetes mellitus (41, 42). Numerous studies have confirmed that renal function decline, defined as eGFR <60 mL/min/1.73 m^2^, was independently associated with an increased risk of cardiovascular events in patients with diabetes (43–45). We found that the incidence and severity of CVD in patients with eGFR of 60–84.8 mL/min/1.73 m^2^ was higher than in patients with eGFR >84.8 mL/min/1.73 m^2^, so we suggested that the range of 60-84.8 ml/min/1.73 m^2^ of eGFR may already increase the risk for CVD. Albuminuria is believed to reflect endothelial injury that extends from the glomerulus to the arterial circulation at large, thus linking this marker to both kidney disease and cardiovascular disease (CVD) (46–50). A study showed that participants with UACR median value of ≥3.9 mg/g for men, and ≥7.5 mg/g for women experienced a nearly 3-fold higher risk of CVD than those with UACR below the median (51). Consistent with this study, we also found that the incidence and severity of CVD were significantly higher in high-risk patients (UACR at 15.5-30 mg/g) than in low-risk patients (UACR <15.5 mg/g). This adds to the growing body of evidence that challenges the notion that UACR <30 mg/g indicates “normal” albumin excretion.

We have several limitations. First, our samples size was not large enough. To overcome this, we randomly selected patients from three tertiary hospital in the validationn cohort. This may enhance the effectiveness of the study. However, a more comprehensive analysis of the relationship between eGFR ≤84.8 mL/min/1.73 m2 or UACR ≥15.5 mg/g and CVD are needed. Secondly, we did not obtain the general data such as blood biochemistry except UACR and eGFR after follow-up. Our focus was to study the impact of the general data of the baseline on the outcome in order to find the risk factors leading to DKD. Therefore, we did not record the general data of the patient at the end of follow-up except UACR and eGFR. In addition, we believe that the general data at the end of follow-up and the outcome events occur at the same time, therefore they may have limited impact on our research results.

In conclusion, eGFR ≤84.8 mL/min/1.73 m^2^ or UACR ≥15.5 mg/g in the normal range may be an effective cut-off value for DKD. Paying attention to the decrease of eGFR and increase of UACR within the normal range and providing early and reasonable intervention may prevent or delay the development of DKD. In addition, patients who had eGFR lower than or equal to the cut-off value or UACR higher than or equal to the cut-off value may had higher incidence and severity for CVD.

## Data availability statement

The raw data supporting the conclusions of this article will be made available by the authors, without undue reservation.

## Ethics statement

This study was approved by the Institutional Review Board of Tianjin Medical University Chu Hsien-I Memorial Hospital and Tianjin Institute of Endocrinology. Informed consent was obtained from all patients prior to study participation.

## Author contributions

ZG and YZ performed the research, analyzed the data and wrote the manuscript and they are co-first authors and contributed equally to this study. XS, WJ, MS, JW, LL, HuZ, YQ, SZ, YY, JX performed the research and acquired data. HoZ contributed to statistical analyses. JY, CS and BC designed the study and revised the manuscript, and they are co-corresponding authors and contributed equally to this study, and they take full responsibility for the work and approved the final version to be published. All authors contributed to the article and approved the submitted version.
