# Supplementary material for: Establishment and validation of the cut-off values of estimated glomerular filtration rate and urinary albumin-to-creatinine ratio for diabetic kidney disease: A multi-center, prospective cohort study
Source: Front Endocrinol (Lausanne). 2022 Dec 12;13:1064665. doi: 10.3389/fendo.2022.1064665 (PMC9791215; doi:10.3389/fendo.2022.1064665)
Supplement: Supplementary file 1 [file DataSheet_1.docx]

**Content**

| Supplementary Table 1 |
| --- |
| Supplementary Table 2 |
| Supplementary Table 3  Supplementary Table 4  Supplementary Table 5  Supplementary Table 6  Supplementary Table 7  Supplementary Table 8  Supplementary Table 9  Supplementary Table 10  Supplementary Figure 1 |

Supplementary Table 1 Baseline characteristics of patients with and without DKD in development cohort

| Variables | Total | DKD | | *P* value |
| --- | --- | --- | --- | --- |
|  |  | No | Yes |  |
| n | 374 | 280 | 94 |  |
| Follow-up (months) | 35.22±15.13 | 34.60±14.64 | 35.22±16.08 | 0.728 |
| Age (years) | 54.72±10.42 | 54.23±10.31 | 56.18±10.66 | 0.116 |
| Female (%) | 164 (43.9) | 134 (47.9) | 30 (31.9) | 0.007 |
| Diabetes duration (years) | 7 (3, 12) | 6 (3, 12) | 8 (3, 14) | 0.335 |
| BMI (kg/m^2^) | 26.58±3.61 | 26.67 ± 3.59 | 26.29±3.68 | 0.380 |
| SBP (mmHg) | 130 (120, 140) | 130 (120, 140) | 130 (120, 140) | 0.111 |
| DBP (mmHg) | 80 (70, 85) | 80 (70, 85) | 80 (70, 90) | 0.693 |
| HbA1c (%) | 8.60 (7.30, 9.93) | 8.50 (7.30, 9.87) | 8.90 (7.20, 10.43) | 0.155 |
| TG (mmol/L) | 1.63 (1.12, 2.53) | 1.63 (1.10, 2.53) | 1.66 (1.17, 2.63) | 0.340 |
| HDL-C (mmol/L) | 1.20 (1.10, 1.40) | 1.20 (1.10, 1.40) | 1.20 (1.10, 1.40) | 0.312 |
| TC (mmol/L) | 5.03±1.10 | 5.01±1.06 | 5.11±1.25 | 0.425 |
| LDL-C (mmol/L) | 3.13±0.89 | 3.09±0.85 | 3.24±1.00 | 0.144 |
| SCr (umol/L) | 61.59±12.03 | 60.57±11.72 | 64.62±12.50 | 0.005 |
| SUA (umol/L) | 304.96±79.13 | 298.08±80.15 | 325.17±72.78 | 0.004 |
| Baseline eGFR (ml/min/1.73 m^2^) | 99.35±18.03 | 101.21±17.35 | 93.81± 18.97 | 0.001 |
| Baseline UACR (mg/g) | 14.56 (11.86, 18.49) | 14.00 (11.50, 17.50) | 18.48 (13.46, 26.14) | < 0.001 |
| DR (n (%)) | 95 (25.40) | 55 (19.64) | 40 (42.55) | < 0.001 |
| Oral diabetic medications (n (%)) | 369 (98.66) | 277 (98.93) | 92 (97.87) | 0.440 |
| Insulin (n (%)) | 208 (55.61) | 154 (55.00) | 54 (57.45) | 0.680 |
| ACEI/ARB (n (%)) | 109 (29.14) | 78 (27.86) | 31 (32.98) | 0.344 |
| Statins (n (%)) | 94 (25.5) | 69 (24.6) | 25 (26.6) | 0.706 |

Supplementary Table 2 Baseline characteristics of patients with and without DKD in validation cohort

| Variables | Total | DKD | | *P* value |
| --- | --- | --- | --- | --- |
|  |  | No | Yes |  |
| n | 181 | 149 | 32 |  |
| Follow-up (months) | 36.47±19.54 | 36.08±18.79 | 38.25±22.89 | 0.572 |
| Age (years) | 55.99±8.98 | 55.79±9.08 | 56.91±8.56 | 0.525 |
| Female (%) | 77 (42.5) | 65 (43.6) | 12 (37.5) | 0.449 |
| Diabetes duration (years) | 6 (2, 10) | 6 (2, 10) | 8.5 (3, 12) | 0.277 |
| BMI (kg/m^2^) | 26.34±4.00 | 26.16 ± 3.94 | 27.26±4.26 | 0.314 |
| SBP (mmHg) | 131 (120, 150) | 130 (120, 150) | 141 (128.5, 150) | 0.109 |
| DBP (mmHg) | 80 (70, 90) | 80 (70, 87) | 82 (70, 90) | 0.371 |
| HbA1c (%) | 7.70 (6.80, 9.45) | 7.60 (6.80, 9.30) | 8.25 (7.10, 10.45) | 0.159 |
| TG (mmol/L) | 1.83 (1.28, 2.67) | 1.83 (1.32, 2.61) | 1.81 (1.22, 2.95) | 0.924 |
| HDL-C (mmol/L) | 0.98 (0.81, 1.14) | 1.02 (0.82, 1.14) | 0.94 (0.76, 1.16) | 0.502 |
| TC (mmol/L) | 5.12±1.19 | 5.12±1.18 | 5.13±1.23 | 0.118 |
| LDL-C (mmol/L) | 3.28±0.93 | 3.26±0.95 | 3.34±0.89 | 0.212 |
| SCr (umol/L) | 59.94±15.54 | 57.48±14.25 | 71.08±16.49 | 0.523 |
| SUA (umol/L) | 307.75±78.28 | 304.64±80.78 | 321.83±65.02 | 0.476 |
| Baseline eGFR (ml/min/1.73 m^2^) | 99.35±18.03 | 104.86±14.57 | 93.60± 17.85 | 0.001 |
| Baseline UACR (mg/g) | 12.40 (5.90, 19.35) | 11.55 (5.80, 16.97) | 19.35 (7.01, 25.98) | 0.002 |
| DR (n (%)) | 43 (23.76) | 30 (20.13) | 13 (40.6) | < 0.001 |
| Oral diabetic medications [n (%)] | 177 (97.79) | 146 (97.99) | 31 (96.88) | 0.93 |
| Insulin [n (%)] | 68 (37.57) | 43 (28.86) | 25 (78.13) | 0.61 |
| ACEI/ARB [n (%)] | 54 (29.83) | 36 (24.16) | 18 (56.25) | 0.75 |
| Statins [n (%)] | 45 (24.86) | 30 (20.13) | 15 (46.88) | 0.73 |

Supplementary Table 3 Baseline characteristics of patients with T2DM grouped by the presence or absence of albuminuria in validation cohort

| Variables | Any albuminuria | | *P* value |
| --- | --- | --- | --- |
|  | No | Yes |  |
| n | 150 | 31 |  |
| Follow-up (years) | 3 (2, 4) | 3.5 (2, 5) | 0.20 |
| Age (years) | 55.19±9.85 | 55.73±9.91 | 0.82 |
| Female (%) | 70 (46.67) | 12 (38.71) | 0.11 |
| Diabetes duration (years) | 10 (7, 13) | 10 (3.8, 12.3) | 0.42 |
| BMI (kg/m^2^) | 26.16±3.95 | 27.27±4.26 | 0.31 |
| SBP (mmHg) | 120 (120, 130) | 130 (120, 140) | 0.01 |
| DBP (mmHg) | 80 (70, 82) | 80 (70, 90) | 0.45 |
| HbA1c (%) | 8.42±2.05 | 8.83±2.25 | 0.42 |
| TG (mmol/L) | 1.38 (1.02, 2.14) | 1.81 (1.15, 3.41) | 0.23 |
| HDL-C (mmol/L) | 1.29±0.28 | 1.39±0.55 | 0.40 |
| TC (mmol/L) | 5.14±1.63 | 5.36±1.65 | 0.48 |
| LDL-C (mmol/L) | 3.22±0.91 | 3.32±1.02 | 0.68 |
| SCr (umol/L) | 63.29±14.22 | 63.61±12.68 | 0.93 |
| SUA (umol/L) | 299.79±81.83 | 294.83±50.70 | 0.79 |
| Baseline eGFR (ml/min/1.73 m^2^) | 107.79±24.26 | 109.83±25.63 | 0.72 |
| Baseline UACR (mg/g) | 14.84 (12.45, 17.00) | 17.17 (14.30, 23.09) | 0.00 |
| DR [n (%)] | 32 (21.33) | 12 (38.71) | 0.06 |
| Oral diabetic medications [n (%)] | 147 (98.00) | 30 (96.77) | 1.00 |
| Insulin [n (%)] | 69 (46.00) | 16 (51.61) | 0.71 |
| ACEI/ARB [n (%)] | 47 (31.33) | 11 (35.48) | 0.90 |
| Statins [n (%)] | 35 (23.33) | 9 (29.03) | 0.80 |

Data are mean ± SD or n (%)

Supplementary Table 4 Baseline characteristics of patients with T2DM grouped by the eGFR greater than or less than 60 ml/min/1.73 m^2^ in validation cohort

| Variables | eGFR < 60 ml/min/1.73 m^2^ | | *P* value |
| --- | --- | --- | --- |
|  | No | Yes |  |
| n | 156 | 21 |  |
| Follow-up (years) | 3 (2, 4) | 3(2, 4) | 0.13 |
| Age (years) | 55.37±9.03 | 60.62±7.19 | 0.01 |
| Female (%) | 70 (44.87) | 7 (33.33) | 0.36 |
| Diabetes duration (years) | 6 (2, 10) | 10 (4, 12 ) | 0.23 |
| BMI (kg/m^2^) | 26.24±4.16 | 27.42±3.51 | 0.39 |
| SBP (mmHg) | 130 (120, 150) | 140 (129, 150) | 0.20 |
| DBP (mmHg) | 80 (70, 90) | 80 (70, 90) | 0.63 |
| HbA1c (%) | 8.26±2.00 | 8.59±2.34 | 0.49 |
| TG (mmol/L) | 1.88 (1.32, 2.68) | 1.54 (1.17, 2.65) | 0.47 |
| HDL-C (mmol/L) | 1.00±0.23 | 0.99±0.34 | 0.85 |
| TC (mmol/L) | 5.11±1.17 | 5.17±1.31 | 0.84 |
| LDL-C (mmol/L) | 3.26±0.94 | 3.38±0.92 | 0.59 |
| SCr (umol/L) | 57.46±14.13 | 78.37±13.12 | 0.00 |
| SUA (umol/L) | 303.28±79.20 | 340.92±63.27 | 0.04 |
| Baseline eGFR (ml/min/1.73 m^2^) | 105.21±14.38 | 85.05±14.49 | 0.00 |
| Baseline UACR (mg/g) | 12.40 (5.95, 19.27) | 7.95 (3.4, 15.72) | 0.54 |
| DR [n (%)] | 35 (22.44) | 8 (38.10) | 0.17 |
| Oral diabetic medications [n (%)] | 152 (97.44) | 21 (100) | 1.00 |
| Insulin [n (%)] | 59 (37.82) | 9 (42.86) | 0.93 |
| ACEI/ARB [n (%)] | 46 (29.49) | 7 (33.33) | 0.77 |
| Statins [n (%)] | 39 (25.00) | 6 (28.57) | 0.81 |

Data are mean ± SD or n (%)

Supplementary Table 5 The subgroup analysis of UACR in the normal and high-normal group of eGFR at the cut-off value of 84.8 mL/min/1.73 m^2^

|  |  | Baseline UACR < 15.5 mg/g | | | | | Baseline UACR ≥ 15.5 mg/g | | |
| --- | --- | --- | --- | --- | --- | --- | --- | --- | --- |
| Indicator | | | Baseline  eGFR < 85 | Baseline  eGFR ≥ 85 | *P* value |  | Baseline  eGFR < 85 | Baseline  eGFR ≥ 85 | *P* value |
| Endpoint UACR | | | 12.5 (10.5, 14.01) | 12.2 (10.0, 13.5) | 0.18 |  | 18.0 (15.0, 37.28) | 17.6 (16.0, 37.8) | 0.73 |

Supplementary Table 6 The sensitivity and specificity of each age group when the cut-off value was 84.8 ml/min.1.73 m^2^.

| Age | AUC | *P* value | Sensitivity | Specificity |
| --- | --- | --- | --- | --- |
| >61 | 0.829 | 0.001 | 0.76 | 0.90 |
| 55-61 | 0.839 | 0.002 | 0.80 | 0.87 |
| 48-55 | 0.776 | 0.016 | 0.84 | 0.73 |
| <48 | 0.910 | 0.001 | 0.84 | 1.00 |

Supplementary Table 7 The multivariable Cox analysis of DKD including all risk factors and age

|  | B | HR | 95% CI | *P* value |  |
| --- | --- | --- | --- | --- | --- |
| Baseline eGFR (ml/min/1.73 m^2^) | -0.118 | 0.889 | 0.856-0.923 | 0.000 |  |
| Diabetes duration (years) | 0.061 | 1.063 | 1.002-1.128 | 0.044 |  |
| SBP (mmHg) | 0.035 | 1.035 | 1.008-1.063 | 0.011 |  |
| DR | 1.223 | 3.396 | 1.374-8.394 | 0.008 |  |
| Age (years) | 0.005 | 1.005 | 0.962-1.051 | 0.809 |  |

Supplementary Table 8 The multivariable Cox analysis of DKD including age

|  | B | HR | 95% CI | *P* value |  |
| --- | --- | --- | --- | --- | --- |
| Baseline eGFR (ml/min/1.73 m^2^) | -0.096 | 0.909 | 0.880-0.938 | 0.000 |  |
| Age (years) | 0.028 | 1.028 | 0.987-1.070 | 0.180 |  |

Supplementary Table 9 The multivariable Cox analysis of DKD including gender

|  | B | HR | 95% CI | *P* value |  |
| --- | --- | --- | --- | --- | --- |
| Baseline eGFR (ml/min/1.73 m^2^) | -0.103 | 0.902 | 0.869-0.936 | 0.000 |  |
| Gender | 0.260 | 1.296 | 0.258-6.504 | 0.752 |  |

Supplementary Table 10 The multivariable Cox analysis of DKD including age and gender

|  | B | HR | 95% CI | *P* value |  |
| --- | --- | --- | --- | --- | --- |
| Baseline eGFR (ml/min/1.73 m^2^) | -0.093 | 0.912 | 0.875-0.949 | 0.000 |  |
| Age (years) | 0.030 | 1.030 | 0.986-1.077 | 0.185 |  |
| Gender | -0.216 | 0.805 | 0.141-4.586 | 0.807 |  |

Supplementary Figure 1 The ROC of each age group when the cut-off value was 84.8 ml/min.1.73 m^2^


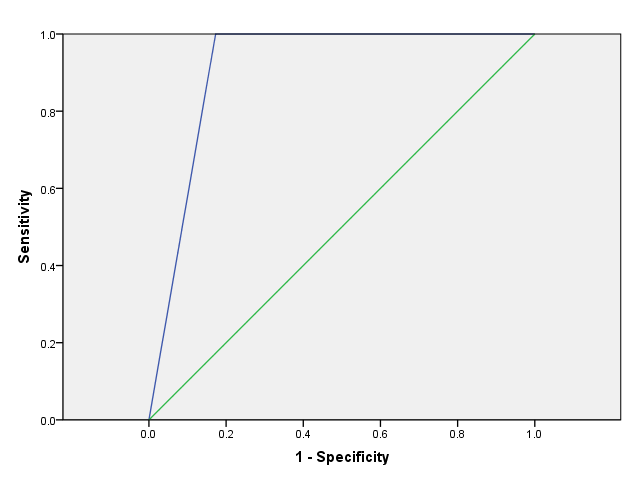

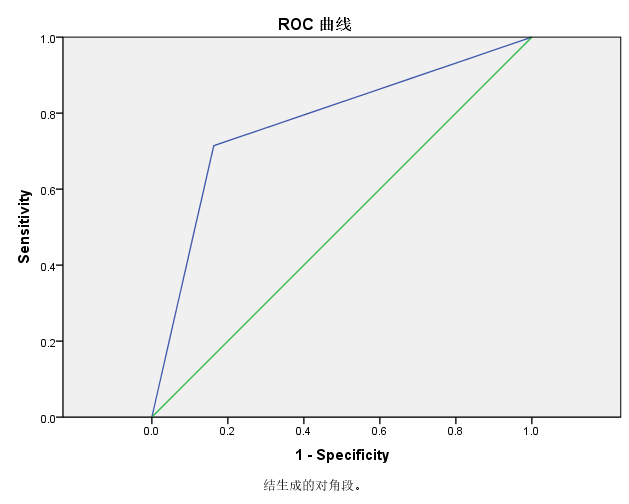

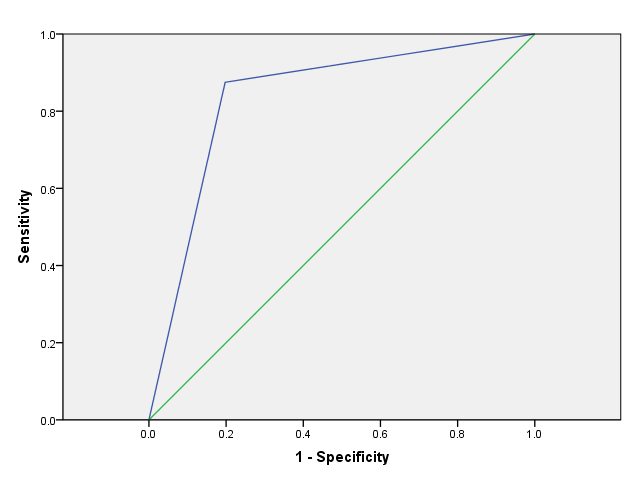

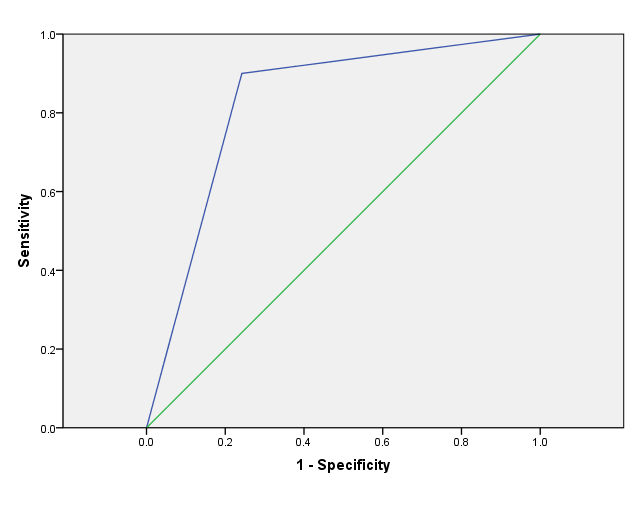


<48

48-55

55-61

>61
